# Prognostic value of triglyceride‐derived metabolic parameters for micro‐ and macrovascular complications and mortality in individuals with type 2 diabetes: The Rio de Janeiro type 2 diabetes cohort study

**DOI:** 10.1111/dme.70263

**Published:** 2026-02-19

**Authors:** Claudia R. L. Cardoso, Guilherme P. Castro, Nathalie C. Leite, Gil F. Salles

**Affiliations:** ^1^ Department of Internal Medicine, School of Medicine, University Hospital Clementino Fraga Filho Universidade Federal do Rio de Janeiro Rio de Janeiro Brazil

**Keywords:** cardiovascular events, cohort study, microvascular complications, mortality, triglyceride‐derived metabolic parameters, type 2 diabetes

## Abstract

**Aims:**

Triglyceride‐derived metabolic parameters have been proposed as indirect measures of insulin resistance and also as predictors of worse prognosis, mainly in Asian populations. However, their value as risk predictors of micro‐ and macrovascular complications in non‐Asian individuals with type 2 diabetes is uncertain.

**Methods:**

Triglyceride‐derived parameters, the atherogenic index of plasma (AIP, the triglyceride/HDL‐cholesterol ratio), the triglyceride‐glucose index (TyG, the triglyceride‐fasting glucose product) and the TyG*BMI were calculated at baseline and during the 1st year of follow‐up in a prospective cohort of 667 individuals with type 2 diabetes. Multivariable Cox analyses assessed the associations between triglyceride‐derived parameters (as continuous and categorical tertile variables) and cardiovascular (total and major cardiovascular events) and microvascular (renal, retinopathy and peripheral neuropathy events) outcomes and mortality.

**Results:**

Over a median 10.6 years of follow‐up, there were 212 total cardiovascular events (172 major ones), 263 all‐cause deaths and 124 new microalbuminuria developments, 98 advanced renal function deteriorations, 154 retinopathy and 173 peripheral neuropathy development/progression events. None of the triglyceride‐derived metabolic parameters, either analysed as continuous or categorical variables, were associated with significantly higher risks for any of the adverse outcomes. The best predictive performance was the 1st year TyG for retinopathy development/progression (HR: 1.24; 95%CI: 1.01–1.53, for increments of 1‐SD), which attenuated to non‐significant (HR: 1.16; 95%CI: 0.92–1.45) after further adjustment for serum LDL‐cholesterol levels.

**Conclusions:**

No triglyceride‐derived metabolic parameter was predictive of any adverse outcome, either micro‐ or macrovascular events or mortality, suggesting that they should not be used for risk stratification in individuals with type 2 diabetes.

## Graphical abstract

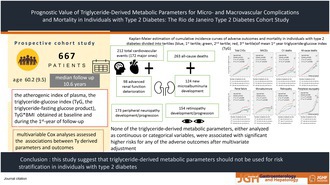

**What's new?**

- Triglyceride‐derived metabolic parameters have been proposed as indirect measures of insulin resistance and also as predictors of worse prognosis, mainly in Asian populations.
- However, triglyceride‐derived metabolic parameters have uncertain value as risk predictors in non‐Asian individuals with type 2 diabetes.
- No triglyceride‐derived metabolic parameter was predictive of either micro‐ or macrovascular events, or mortality.
- They should not be recommended to improve risk stratification in non‐Asian individuals with type 2 diabetes.

## 1 INTRODUCTION

Type 2 diabetes is a highly‐prevalent chronic condition characterized by hyperglycaemia due to insulin resistance (IR) and reduced pancreatic insulin secretion, and is associated with several atherosclerotic macrovascular (coronary, cerebrovascular and peripheral artery diseases) and microvascular (diabetic kidney disease, retinopathy and peripheral neuropathy) complications.^1^, ^2^, ^3^ Worldwide, more than 800 million adult people live with diabetes, 90–95% of them with type 2 diabetes.^4^ IR plays a pivotal role in type 2 diabetes physiopathological mechanisms^5^; however, its gold‐standard measure, the hyperinsulin‐euglycaemic clamp, is invasive, costly, complex and time‐consuming, making it impracticable to routine clinical care use and to large epidemiological studies.^6^ Yet, other established indices of IR, such as the homeostasis model assessment of insulin resistance (HOMA‐IR) and the adipose insulin resistance index (Adipo‐IR) need specific laboratory measurements as serum immunoreactive insulin and free fatty acids levels, which may not be available in routine clinical care. Hence, several surrogate indices of IR derived from serum triglyceride levels were proposed in the past decades, such as the triglyceride‐to‐HDL‐cholesterol ratio, called the atherogenic index of plasma (AIP),^7^ and the triglyceride‐to‐fasting glucose product (TyG),^8^ both calculated on a logarithmic scale. The TyG has also been indexed to measures of body adiposity, such as to body mass index (TyG‐BMI) or to waist circumference (TyG‐WC). These indirect indices of IR have been demonstrated to predict type 2 diabetes incidence and cardiovascular diseases, mainly coronary disease, in general populations, although most of the cohort studies came from Asian populations.^9^, ^10^, ^11^, ^12^ Otherwise, there were scarce studies in individuals with type 2 diabetes, mainly in non‐Asian populations,^6^, ^13^, ^14^, ^15^, ^16^ and with controversial findings. For example, a post‐hoc analysis of the ACCORD trial^13^ and a study from the NHANES database^15^ indicated that the AIP and TyG may predict future cardiovascular events in people with type 2 diabetes, whereas analyses from the Look AHEAD trial^14^ and from the UK Biobank database^6^ suggested that they were not significant predictors of cardiovascular outcomes. Moreover, few longitudinal studies^17^, ^18^ addressed whether these indices were also associated with higher risks of diabetic microvascular complications development. Furthermore, as far as we know, there is no previous cohort study from the Latin America that evaluated these triglyceride‐derived metabolic parameters.

Therefore, this prospective longitudinal study, the Rio de Janeiro Type 2 Diabetes Cohort, with a mixed Brazilian population, aimed to comprehensively assess the prognostic value of several triglyceride‐derived metabolic parameters, namely the AIP, the TyG and the TyG‐BMI, for cardiovascular and microvascular outcomes and for mortality in individuals with type 2 diabetes.

## 2 METHODS

### 2.1 Cohort overview and baseline protocol

This was a prospective longitudinal observational cohort study with 692 individuals with type 2 diabetes, enrolled between 2004 and 2008 and regularly followed up until 2019 in the diabetes outclinic of our tertiary‐care Clementino Fraga Filho University Hospital. All participants gave written informed consent, and the local Ethics Committee had previously approved the study protocol. The characteristics of this cohort, the baseline procedures and the diagnostic definitions have been described previously,^19^, ^20^, ^21^, ^22^, ^23^ and are also on Data S1. For this specific analysis of triglyceride‐derived metabolic parameters, 25 individuals (4%) with baseline serum triglycerides ≥500 mg/dL, possibly indicating familial hypertriglyceridemia, were excluded, totalling 667 participants in this report. All were submitted to a standard baseline protocol that included a thorough clinical laboratory evaluation. Diagnostic criteria for diabetic chronic complications were detailed previously^19^, ^20^, ^21^, ^22^, ^23^ (Data S1). Laboratory evaluation included fasting glycaemia, glycated haemoglobin (HbA_1c_), serum creatinine and lipids. Albuminuria was evaluated in two non‐consecutive sterile 24‐h urine collections.

### 2.2 Triglyceride‐derived metabolic parameters

Three triglyceride‐derived parameters were calculated, as previously described^7^, ^8^: the atherogenic index of plasma (AIP): Log_10_ (Triglyceride (mmol/L)/HDL‐cholesterol (mmol/L)); and the triglyceride‐glucose index (TyG): Ln [(Triglyceride (mg/dL) * fasting glucose (mg/dL))/2]. Additionally, the TyG was also related to body mass index (TyG‐BMI) as TyG * BMI (kg/m^2^).^12^ These parameters were measured at baseline and also as mean values during the first year of follow‐up, where the greatest changes occurred.

### 2.3 Follow‐up and outcomes assessment

The participants were followed up regularly at least 3–4 times a year until December 2019 under standardized treatment. The observation period was considered as the number of months from cohort entry to the date of the last clinical visit in 2019 or the first endpoint, whichever came first. The primary outcomes were the occurrence of any macrovascular or microvascular events and all‐cause mortality. Macrovascular outcomes were total cardiovascular events (CVEs: fatal or non‐fatal myocardial infarctions [MIs], sudden cardiac deaths, new‐onset heart failure, death from progressive heart failure, any myocardial revascularization procedure, fatal or non‐fatal strokes, any aortic or lower limb revascularization procedure, any amputation above the ankle, and deaths from aortic or peripheral arterial disease), major adverse cardiovascular events (MACEs: non‐fatal MIs and strokes plus cardiovascular deaths) and cardiovascular mortality.^22^, ^23^ Cardiovascular and mortality outcomes were adjudicated from medical records (most non‐fatal and fatal in‐hospital events were attended at our hospital), death certificates and interviews with attending physicians and patient families, by a standard questionnaire reviewed by two independent observers.^22^, ^23^ Microvascular outcomes were retinopathy development or worsening,^20^ renal outcomes (new microalbuminuria development, and renal function deterioration, defined as doubling of serum creatinine or end‐stage renal failure needing dialysis or death from renal failure)^21^ and peripheral neuropathy development or worsening.^19^ Microvascular outcomes were evaluated by annual specific examinations. The definitions of retinopathy and peripheral neuropathy development/worsening during follow‐up are detailed in the Data S1.

### 2.4 Statistical analyses

Continuous data were described as means (SD) or as medians (interquartile range [IQR]). Triglyceride‐derived parameters were analysed both as a continuous variable and categorized into tertile subgroups. Baseline clinic‐laboratorial characteristics of participants divided into these tertile subgroups were compared by ANOVA, Kruskall–Wallis or chi‐squared tests, as appropriate. Kaplan–Meier curves of cumulative endpoint incidence during follow‐up, compared by log‐rank tests, were performed for estimating different incidences of outcomes among the tertile subgroups. To address whether the associations between triglyceride‐derived metabolic parameters and adverse outcomes were mainly linear or non‐linear, we performed an initial exploratory time‐to‐event Cox analysis of individuals divided into quantiles (tertiles and quartiles) and also a Cox analysis with restricted cubic splines (knots at the 5th, 35th, 65th and 95th percentile values). In none of these initial analyses, there were evidences of non‐linear associations between any triglyceride‐derived parameter and any of the outcomes (all non‐linear terms *p*‐value >0.20). Hence, to assess the associations between each triglyceride‐derived metabolic parameter and each adverse outcome, a traditional multivariable time‐to‐event Cox analysis was performed with progressively increasing adjustments. Model 1 was only adjusted for age and sex; Model 2 was further adjusted for other potential confounding risk factors: BMI (except in TyG‐BMI analyses, and with body height instead of BMI in neuropathy analyses), smoking, physical activity, diabetes duration, pre‐existent macro‐ and microvascular complications, baseline SBP and HbA_1c_, number of anti‐hypertensive drugs in use, and use of insulin, statins, fibrates and aspirin (renal outcomes were further adjusted for baseline eGFR and albuminuria), and Model 3 was further adjusted for serum LDL‐cholesterol or total cholesterol. In analyses of mean 1st year triglyceride parameters, BMI, SBP, HbA_1c_ and LDL‐ and total cholesterol were also updated to mean 1st year values. Results were presented as hazard ratios (HRs) with their 95% confidence intervals (CIs). To allow comparisons among the triglyceride parameters, HRs in analyses with continuous variables were standardized for increments of 1‐SD in each parameter. In analyses of triglyceride parameters as categorical variables, the upper tertile subgroup was compared with the reference lower tertile subgroup. Statistics were performed with SPSS version 19.0 (SPSS Inc., Chicago, Il., USA) and R version 3.4.1 (R Foundation for Statistical Computing, Vienna, Austria), and a 2‐tailed probability value <0.05 was considered significant.

## 3 RESULTS

### 3.1 Baseline characteristics of participants

Table 1 presents the baseline clinic‐laboratorial characteristics of the 667 individuals with type 2 diabetes evaluated and of those divided according to tertiles of baseline AIP. Participants at the higher tertile subgroup were more obese with greater waist circumference and had a greater prevalence of chronic kidney disease (reduced eGFR and increased albuminuria). They also had a greater prevalence of dyslipidaemia, with more frequent use of fibrates (but not of statins), and higher serum total cholesterol levels, beyond the expected differences in triglyceride and HDL‐cholesterol levels. Table 2 presents the same characteristics of participants divided according to tertiles of baseline TyG index. By contrast to AIP, individuals at the higher tertile subgroup were younger, less frequently males, used insulin more frequently and had higher BP levels at baseline. Similar to AIP strata, they were more obese, with greater waist circumferences, and had a greater prevalence of chronic kidney disease. They also had a greater prevalence of dyslipidaemia, with a greater use of fibrates, and higher serum levels of all lipid fractions, including total and LDL‐cholesterol, both at baseline and during the first year of follow‐up, beyond the expected worse glycaemic control.

**TABLE 1:** Clinical‐laboratory characteristics of all 667 individuals with type 2 diabetes and divided into tertiles of baseline atherogenic index of plasma (AIP).

Characteristics	All patients (*n* = 667)	1st‐tertile AIP < 0.036 (*n* = 222)	2nd‐tertile AIP 0.036–0.317 (*n* = 223)	3rd‐tertile AIP > 0.317 (*n* = 222)	*p*‐value
Age (years)	60.2 (9.5)	60.4 (9.3)	60.6 (9.1)	59.6 (10.3)	0.53
Man (%)	38.7	39.1	37.8	39.1	0.95
Body mass index (kg/m^2^)	29.7 (4.8)	28.8 (4.9)	30.1 (4.9)*	30.2 (4.5)*	0.002
Waist circumference (cm)	102 (11)	100 (12)	103 (11)*	104 (10)*	<0.001
Smoking, current/past (%)	44.2	40.0	44.3	48.8	0.18
Physical activity (%)	22.3	21.3	25.2	20.3	0.42
Diabetes duration (years)	8 (3–15)	9.5 (4–16)	8 (3–14)	8 (2.5–15)	0.19
Chronic diabetic complications (%)					
Cerebrovascular disease	8.8	9.1	5.7	12.1	0.06
Coronary artery disease	15.1	12.2	14.8	18.8	0.15
Peripheral artery disease	17.0	13.5	17.4	20.4	0.16
Retinopathy	33.1	36.0	29.9	33.3	0.39
Nephropathy	31.1	24.1	31.3	38.8*	0.005
Peripheral neuropathy	29.2	26.5	32.4	28.6	0.37
Diabetes treatment (%)					
Metformin	87.6	85.7	89.6	87.4	0.45
Sulfonylureas	42.6	40.0	42.2	45.9	0.46
Insulin	48.9	47.8	49.6	49.3	0.92
Other medications					
Aspirin	89.5	87.4	89.6	91.8	0.33
Dyslipidaemia (%)	86.7	79.1	84.8	97.1*	<0.001
Statins use (%)	77.7	73.9	81.7	77.3	0.13
Fibrates use (%)	3.7	0	0.4	11.6*	<0.001
Arterial hypertension (%)	86.5	83.9	89.0	86.6	0.29
Number of anti‐hypertensive drugs	3 (1–3)	2 (1–3)	3 (1–3)	3 (1–3)	0.52
ACE inhibitors/AR blockers (%)	81.4	77.8	86.5	79.7	0.042
Diuretics (%)	62.4	57.0	65.2	65.2	0.11
Calcium channel blockers (%)	28.0	29.1	27.4	27.5	0.90
Beta‐blockers (%)	45.6	39.1	47.8	50.2	0.046
Blood pressures (mmHg)					
Baseline clinic SBP	147 (24)	144 (25)	147 (24)	149 (23)	0.13
Baseline clinic DBP	84 (13)	82 (12)	85 (14)	85 (13)	0.041
Mean 1st year SBP	140 (19)	140 (21)	140 (18)	141 (18)	0.78
Mean 1st year DBP	79 (11)	79 (11)	79 (11)	80 (11)	0.29
Laboratory variables					
Baseline FG (mmol/L)	8.9 (3.8)	8.6 (3.7)	8.6 (3.6)	9.4 (4.1)*	0.033
Mean 1st year FG (mmol/L)	8.1 (2.8)	7.8 (2.6)	7.9 (2.8)	8.6 (3.1)*	0.007
Baseline HbA_1c_ (mmol/mol)	64 (21)	65 (22)	64 (20)	64 (20)	0.60
(%)	8.0 (1.9)	8.1 (2.0)	8.0 (1.8)	8.0 (1.8)	
Mean 1st year HbA_1c_ (mmol/mol)	61 (18)	61 (18)	61 (18)	61 (16)	0.95
(%)	7.7 (1.6)	7.7 (1.6)	7.7 (1.6)	7.7 (1.5)	
Baseline triglycerides (mmol/L)	1.82 (0.98)	1.00 (0.28)	1.62 (0.38)*	2.96 (0.90)*	<0.001
Mean 1st year triglycerides (mmol/L)	1.74 (0.98)	1.08 (0.40)	1.60 (0.56)*	2.64 (1.12)*	<0.001
Baseline total cholesterol (mmol/L)	5.0 (1.2)	4.8 (1.2)	4.9 (1.1)	5.4 (1.1)*	<0.001
Mean 1st year total cholesterol (mmol/L)	4.8 (1.0)	4.6 (1.0)	4.7 (0.9)	5.0 (1.1)*	<0.001
Baseline HDL‐cholesterol (mmol/L)	1.1 (0.3)	1.3 (0.3)	1.1 (0.2)*	0.9 (0.2)*	<0.001
Mean 1st year HDL‐cholesterol (mmol/L)	1.1 (0.3)	1.3 (0.3)	1.1 (0.2)*	1.0 (0.2)*	<0.001
Baseline LDL‐cholesterol (mmol/L)	3.1 (1.0)	2.9 (1.0)	3.1 (1.0)	3.2 (1.1)	0.082
Mean 1st year LDL‐cholesterol (mmol/L)	2.8 (0.9)	2.8 (0.9)	2.8 (0.8)	2.8 (0.9)	0.67
Glomerular filtration rate (mL/min/1.73 m^2^)	81 (20)	86 (18)	80 (19)*	76 (22)*	<0.001
Albuminuria (mg/24 h)	13 (7–38)	12 (7–25)	13 (7–38)	15 (8–52)*	0.006
Triglycerides‐derived parameters					
Baseline AIP	0.17 (0.28)	−0.14 (0.14)	0.19 (0.08)*	0.50 (0.14)*	<0.001
Mean 1st year AIP	0.14 (0.28)	−0.11 (0.19)	0.15 (0.15)*	0.41 (0.20)*	<0.001
Baseline TyG	9.25 (0.70)	8.70 (0.54)	9.22 (0.47)*	9.89 (0.52)*	<0.001
Mean 1st year TyG	9.15 (0.66)	8.72 (0.50)	9.10 (0.53)*	9.66 (0.57)*	<0.001
Baseline TyG‐BMI	274.8 (51.6)	250.6 (46.1)	277.5 (48.6)*	298.7 (48.8)*	<0.001
Mean 1st year TyG‐BMI	271.9 (50.4)	252.5 (47.4)	273.9 (49.2)*	292.0 (47.1)*	<0.001

*Note*: Values are proportions, and means (SD) or medians (interquartile range).

Abbreviations: ACE, angiotensin‐converting enzyme; AIP, atherogenic index of plasma; AR, angiotensin II receptor; DBP, diastolic blood pressure; FG, fasting glycaemia; HbA_1c_, glycated haemoglobin; HDL, high‐density lipoprotein; LDL, low‐density lipoprotein; SBP, systolic blood pressure; TyG, triglyceride‐glucose index; TyG‐BMI, triglyceride‐glucose‐body mass index.

* Significant difference (*p* < 0.05 after Bonferroni's correction for multiple comparisons) in relation to the first tertile subgroup.

**TABLE 2:** Clinical‐laboratory characteristics of all 667 individuals with type 2 diabetes and divided into tertiles of baseline triglyceride‐glucose index (TyG).

Characteristics	All individuals (*n* = 667)	1st‐tertile TyG < 8.94 (*n* = 222)	2nd‐tertile TyG 8.94–9.59 (*n* = 223)	3rd‐tertile TyG > 9.59 (*n* = 222)	*p*‐value
Age (years)	60.2 (9.5)	61.0 (9.2)	60.8 (9.7)	58.8 (9.7)*	0.032
Man (%)	38.7	44.5	38.3	32.0*	0.028
Body mass index (kg/m^2^)	29.7 (4.8)	28.9 (4.9)	29.8 (4.9)	30.4 (4.4)*	0.008
Waist circumference (cm)	102 (11)	101 (12)	103 (11)	104 (10)*	0.011
Smoking, current/past (%)	44.2	40.2	47.0	45.1	0.32
Physical activity (%)	22.3	24.5	20.9	21.8	0.64
Diabetes duration (years)	8 (3–15)	8 (3–15)	9 (4–15)	8 (3–15)	0.98
Chronic diabetic complications (%)					
Cerebrovascular disease	8.8	8.7	7.4	10.7	0.48
Coronary artery disease	15.1	14.4	14.3	17.0	0.69
Peripheral artery disease	17.0	15.8	17.8	17.6	0.82
Retinopathy	33.1	29.9	34.8	35.0	0.44
Nephropathy	31.1	24.4	26.1	43.8*	<0.001
Peripheral neuropathy	29.2	26.8	27.4	33.5	0.25
Diabetes treatment (%)					
Metformin	87.6	84.3	90.0	88.3	0.16
Sulfonylureas	42.6	35.4	48.3	44.2	0.055
Insulin	48.9	44.5	44.3	59.2*	0.003
Other medications					
Aspirin	89.5	87.8	89.1	92.2	0.30
Dyslipidaemia (%)	86.7	80.3	84.8	95.6*	<0.001
Statins use (%)	77.7	77.3	77.8	78.2	0.96
Fibrates use (%)	3.7	0.4	2.2	9.2*	<0.001
Arterial hypertension (%)	86.5	83.4	87.8	88.8	0.21
Number of anti‐hypertensive drugs	3 (1–3)	2 (1–3)	3 (1–3)	3 (1–3)	0.55
ACE inhibitors/AR blockers (%)	81.4	80.3	82.2	81.6	0.88
Diuretics (%)	62.4	56.3	65.7	65.5	0.064
Calcium channel blockers (%)	28.0	32.8	25.7	25.7	0.16
Beta‐blockers (%)	45.6	35.8	48.3	53.4*	0.001
Blood pressures (mmHg)					
Baseline clinic SBP	147 (24)	145 (25)	145 (24)	150 (25)*	0.043
Baseline clinic DBP	84 (13)	82 (13)	83 (12)	87 (14)*	0.003
Mean 1st year SBP	140 (19)	140 (20)	139 (19)	142 (18)	0.21
Mean 1st year DBP	79 (11)	78 (11)	79 (11)	81 (10)*	0.022
Laboratory variables					
Baseline FG (mmol/L)	8.9 (3.8)	6.6 (2.1)	8.4 (2.5)*	11.9 (4.4)*	<0.001
Mean 1st year FG	8.1 (2.8)	7.3 (2.2)	7.7 (2.4)	9.4 (3.3)*	<0.001
Baseline HbA_1c_ (mmol/mol)	64 (21)	58 (16)	63 (20)	73 (23)	
(%)	8.0 (1.9)	7.5 (1.5)	7.9 (1.8)*	8.8 (2.1)*	<0.001
Mean 1st year HbA_1c_ (mmol/mol)	61 (18)	56 (15)	58 (15)	67 (20)	
(%)	7.7 (1.6)	7.3 (1.4)	7.5 (1.4)	8.3 (1.8)*	<0.001
Baseline triglycerides (mmol/L)	1.82 (0.98)	1.06 (0.40)	1.72 (0.52)	2.78 (1.02)	<0.001
Mean 1st year triglycerides (mmol/L)	1.74 (0.98)	1.16 (0.50)	1.64 (0.80)*	2.50 (1.10)*	<0.001
Baseline total cholesterol (mmol/L)	5.0 (1.2)	4.6 (1.0)	5.0 (1.1)*	5.6 (1.2)*	<0.001
Mean 1st year total cholesterol (mmol/L)	4.8 (1.0)	4.5 (1.0)	4.6 (1.0)	5.2 (1.0)*	<0.001
Baseline HDL‐cholesterol (mmol/L)	1.1 (0.3)	1.2 (0.3)	1.1 (0.3)*	1.0 (0.3)*	<0.001
Mean 1st year HDL‐cholesterol (mmol/L)	1.1 (0.3)	1.2 (0.3)	1.1 (0.3)*	1.0 (0.3)*	<0.001
Baseline LDL‐cholesterol (mmol/L)	3.1 (1.0)	2.9 (0.9)	3.1 (1.0)	3.2 (1.1)*	0.004
Mean 1st year LDL‐cholesterol (mmol/L)	2.8 (0.9)	2.7 (0.9)	2.7 (0.8)	2.9 (0.9)*	0.016
Glomerular filtration rate (mL/min/1.73 m^2^)	81 (20)	84 (19)	82 (19)	77 (22)*	0.003
Albuminuria (mg/24 h)	13 (7–38)	12 (7–24)	12 (7–27)	17 (8–108)*	<0.001
Triglyceride‐derived parameters					
Baseline AIP	0.17 (0.28)	−0.07 (0.21)	0.19 (0.19)*	0.42 (0.22)*	<0.001
Mean 1st year AIP	0.14 (0.28)	−0.05 (0.22)	0.15 (0.22)*	0.35 (0.24)*	<0.001
Baseline TyG	9.25 (0.70)	8.51 (0.36)	9.26 (0.19)*	10.06 (0.37)*	<0.001
Mean 1st year TyG	9.15 (0.66)	8.70 (0.50)	9.09 (0.50)*	9.69 (0.56)*	<0.001
Baseline TyG‐BMI	274.8 (51.6)	246.3 (44.2)	275.6 (46.0)*	305.5 (47.1)*	<0.001
Mean 1st year TyG‐BMI	271.9 (50.4)	251.3 (46.9)	271.6 (48.5)*	294.6 (46.7)*	<0.001

*Note*: Values are proportions, and means (standard deviations) or medians (interquartile range).

Abbreviations: ACE, angiotensin‐converting enzyme; AIP, atherogenic index of plasma; AR, angiotensin II receptor; DBP, diastolic blood pressure; FG, fasting glycaemia; HbA_1c_, glycated haemoglobin; HDL, high‐density lipoprotein; LDL, low‐density lipoprotein; SBP, systolic blood pressure; TyG, triglyceride‐glucose index; TyG‐BMI, triglyceride‐glucose‐body mass index.

* Significant difference (*p* < 0.05 after Bonferroni's correction for multiple comparisons) in relation to the first tertile subgroup.

### 3.2 Incidence of adverse outcomes

**FIGURE 1:**
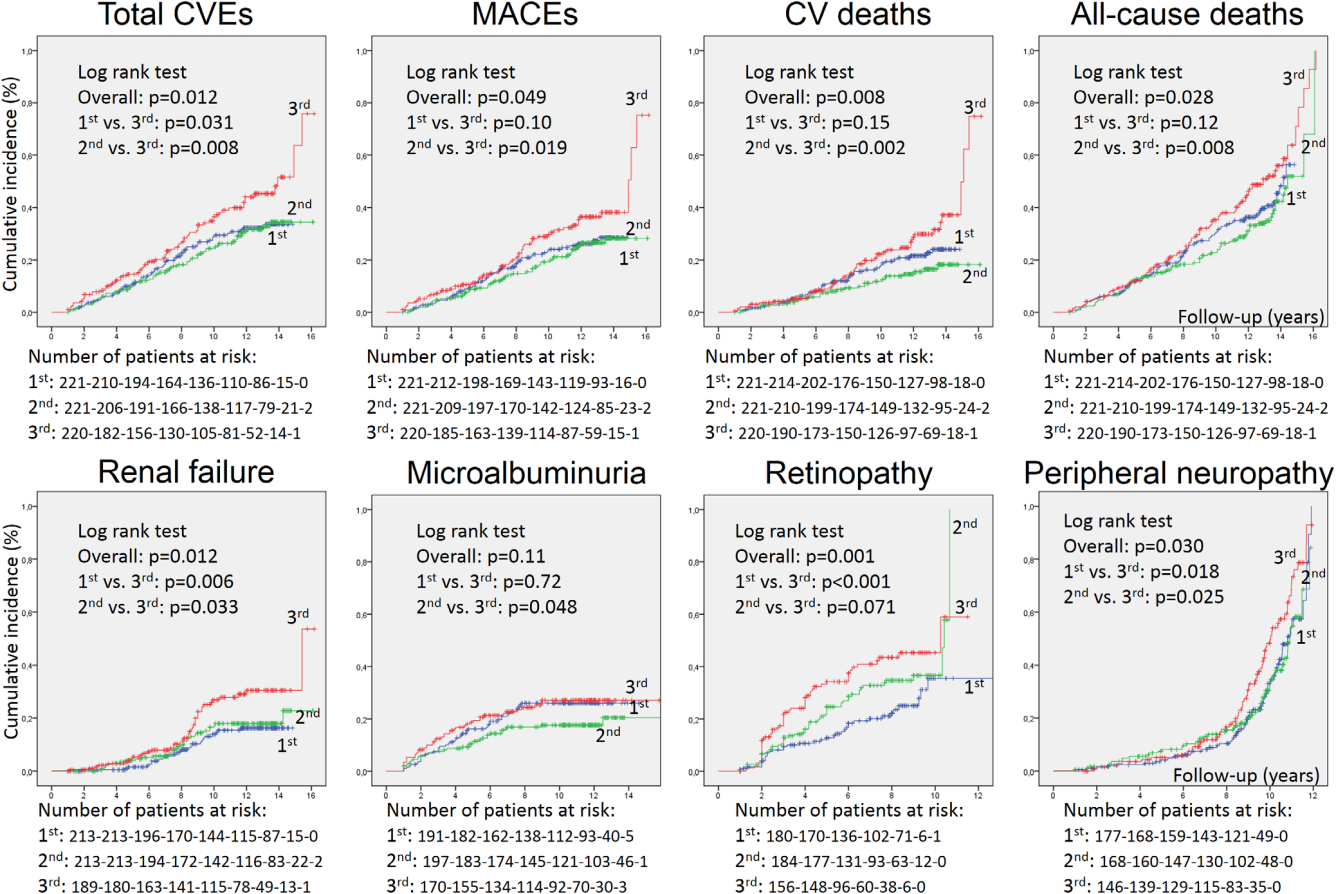
Kaplan–Meier estimation of cumulative incidence curves of adverse macro‐ and microvascular outcomes and mortality in individuals with type 2 diabetes divided into tertiles (blue, 1st tertile; green, 2nd tertile; red, 3rd tertile) of mean 1st year triglyceride‐glucose index (TyG). Overall and pairwise comparisons among subgroups were performed by log‐rank tests. CVEs, cardiovascular events; MACEs, major adverse cardiovascular events.

Over a median follow‐up of 10.6 years (IQR: 6.3 to 13.1 years, maximum 16.2 years), corresponding to 6347 person‐years of follow‐up, there were 212 total CVEs (172 MACEs) and 263 all‐cause deaths (129 from cardiovascular diseases), 124 new microalbuminuria development, 98 advanced renal function deterioration, 154 retinopathy and 173 peripheral neuropathy development/progression events. Table 3 outlines the incidence rates of these adverse outcomes in individuals divided according to tertiles of AIP, TyG and TyG‐BMI indices measured at baseline and during the 1st year of follow‐up. There were no differences in cumulative incidences of outcomes among tertile subgroups when the triglyceride‐derived parameters were measured at baseline, except in the upper tertile of TyG for advanced renal failure. Otherwise, when measured as mean values during the 1st year, there were higher incidences of most adverse outcomes in the upper tertile subgroup of TyG, except for microalbuminuria development. The upper tertile subgroup of 1st year AIP had only a greater incidence of advanced renal failure, whereas the upper tertile subgroup of 1st year TyG‐BMI had only a greater incidence of peripheral neuropathy events. The Kaplan–Meier curves of cumulative incidences of adverse outcomes in participants divided into tertiles of mean 1st year TyG are shown in Figure 1.

**TABLE 3:** Incidences of macro‐ and microvascular outcomes and mortality during follow‐up in all 667 participants with type 2 diabetes and divided according to tertile subgroups of triglyceride‐derived metabolic parameters measured at baseline and during the first year of follow‐up.

Outcomes	All participants	Tertiles of triglyceride‐derived parameters			*p*‐value^a^
		1st Tertile	2nd Tertile	3rd Tertile	
Baseline AIP					
Total CVEs	212 (36.1)	65 (31.8)	79 (38.7)	68 (38.0)	0.51
MACEs	172 (28.2)	53 (25.0)	66 (31.5)	53 (28.2)	0.50
CV mortality	129 (20.3)	39 (17.9)	47 (21.5)	43 (21.8)	0.71
All‐cause mortality	263 (41.4)	84 (38.5)	91 (41.5)	88 (44.5)	0.87
Advanced renal failure	98 (16.5)	33 (16.2)	27 (13.0)	38 (20.8)	0.17
Microalbuminuria	124 (25.9)	48 (30.1)	40 (23.8)	36 (23.7)	0.42
Retinopathy	154 (50.5)	52 (50.0)	57 (54.9)	45 (47.4)	0.79
Peripheral neuropathy	173 (43.1)	56 (40.6)	59 (44.3)	58 (46.0)	0.84
Baseline TyG					
Total CVEs	212 (36.1)	73 (35.8)	70 (33.4)	69 (40.0)	0.55
MACEs	172 (28.2)	62 (29.5)	57 (25.9)	53 (29.7)	0.66
CV mortality	129 (20.3)	43 (19.7)	44 (19.4)	42 (22.3)	0.77
All‐cause mortality	263 (41.4)	85 (38.8)	89 (39.3)	89 (47.2)	0.36
Renal failure	98 (16.5)	30 (14.7)	28 (13.0)	40 (23.1)*	0.031
Microalbuminuria	124 (25.9)	48 (30.0)	35 (19.8)	41 (28.7)	0.18
Retinopathy	154 (50.5)	47 (43.2)	54 (48.7)	53 (62.5)	0.14
Peripheral neuropathy	173 (43.1)	54 (38.4)	54 (38.0)	65 (54.9)	0.15
Baseline TyG‐BMI					
Total CVEs	212 (36.1)	74 (37.0)	68 (32.8)	70 (39.2)	0.53
MACEs	172 (28.2)	64 (30.7)	54 (25.2)	54 (29.1)	0.50
CV mortality	129 (20.3)	42 (19.2)	43 (19.2)	44 (23.0)	0.55
All‐cause mortality	263 (41.4)	92 (42.1)	85 (38.0)	86 (44.9)	0.37
Renal failure	98 (16.5)	33 (16.0)	32 (15.3)	33 (18.6)	0.60
Microalbuminuria	124 (25.9)	42 (25.5)	49 (29.5)	33 (22.3)	0.63
Retinopathy	154 (50.5)	54 (51.2)	54 (49.1)	46 (51.6)	0.93
Peripheral neuropathy	173 (43.1)	51 (37.4)	63 (43.6)	59 (49.0)	0.25
Mean 1st year AIP					
Total CVEs	206 (35.9)	64 (32.1)	77 (38.4)	65 (38.1)	0.56
MACEs	168 (28.2)	52 (25.5)	59 (28.3)	57 (31.4)	0.61
CV mortality	128 (20.6)	41 (18.8)	41 (18.7)	46 (24.0)	0.44
All‐cause mortality	260 (41.9)	84 (39.0)	86 (38.6)	90 (47.5)	0.41
Renal failure	97 (16.6)	30 (15.2)	25 (11.6)	42 (24.0)*	0.008
Microalbuminuria	123 (26.0)	47 (30.0)	39 (22.0)	37 (26.0)	0.32
Retinopathy	150 (49.7)	49 (47.8)	49 (45.4)	52 (60.0)	0.29
Peripheral neuropathy	171 (43.1)	59 (43.9)	56 (38.9)	56 (47.1)	0.73
Mean 1st year TyG					
Total CVEs	206 (35.9)	64 (31.3)	64 (31.1)	78 (47.7)*	0.012
MACEs	168 (28.2)	53 (25.1)	52 (24.5)	63 (36.7)	0.049
CV mortality	128 (20.6)	42 (19.3)	34 (15.5)	52 (28.3)*	0.008
All‐cause mortality	260 (41.9)	84 (38.6)	81 (37.0)	95 (51.7)*	0.028
Renal failure	97 (16.6)	26 (12.5)	31 (14.8)	40 (24.0)*	0.012
Microalbuminuria	123 (26.0)	47 (28.7)	33 (19.0)	43 (31.5)	0.11
Retinopathy	150 (49.7)	37 (32.8)	56 (51.5)*	57 (70.9)*	0.001
Peripheral neuropathy	171 (43.1)	56 (38.7)	51 (37.5)	64 (55.2)*	0.030
Mean 1st year TyG‐BMI					
Total CVEs	196 (34.5)	65 (33.6)	64 (32.2)	67 (38.1)	0.55
MACEs	160 (27.3)	56 (27.9)	52 (25.5)	52 (28.5)	0.78
CV mortality	119 (19.4)	38 (18.3)	37 (17.2)	44 (23.3)	0.32
All‐cause mortality	249 (40.7)	93 (44.8)	77 (35.8)	79 (41.7)	0.23
Renal failure	95 (16.4)	32 (16.2)	26 (12.6)	37 (21.2)	0.076
Microalbuminuria	123 (26.2)	42 (26.6)	48 (29.1)	33 (22.5)	0.66
Retinopathy	148 (49.8)	43 (40.5)	56 (53.7)	49 (56.7)	0.21
Peripheral neuropathy	169 (42.9)	45 (34.3)	58 (41.3)	66 (54.2)*	0.029

*Note*: Values are absolute numbers of events (incidence rate per 1000 person‐years of follow‐up).

^a^ *p*‐values were estimated from log‐rank tests applied to Kaplan–Meier analyses of cumulative incidence curves of outcomes during follow‐up.

* Significant difference (*p* < 0.05 after Bonferroni's correction for multiple comparisons) in relation to the first tertile subgroup.

### 3.3 Adjusted risks for macro‐ and microvascular outcomes and mortality associated with triglyceride‐derived metabolic parameters

Table 4 shows the adjusted HRs associated with triglyceride‐derived parameters, measured at baseline and during the 1st year of follow‐up, analysed as continuous and categorical (tertiles) variables, for macrovascular and mortality outcomes. None of the metabolic parameters were associated with significantly higher risks of cardiovascular and mortality endpoints after adjustment for traditional risk factors, even without including cholesterol‐related factors (Model 2). Indeed, most were non‐significant yet after simple adjustment for age and sex (Model 1).

**TABLE 4:** Adjusted risks associated with triglyceride‐derived metabolic parameters (continuous and categorical), measured at baseline and during the first year of follow‐up, for cardiovascular outcomes and mortality in 667 individuals with type 2 diabetes.

Outcomes	Model 1^a^	Model 2^a^	Model 3^a^
Triglyceride‐derived metabolic parameters	HR (95% CI)	HR (95% CI)	HR (95% CI)
Total cardiovascular events (*n* = 212)			
Baseline continuous AIP	1.08 (0.92–1.26)	1.01 (0.85–1.20)	0.98 (0.81–1.18)
Baseline continuous TyG	1.06 (0.91–1.24)	0.94 (0.80–1.11)	0.89 (0.74–1.07)
Baseline continuous TyG‐BMI	1.04 (0.90–1.22)	0.93 (0.79–1.09)	0.91 (0.77–1.07)
Baseline upper tertile AIP	1.18 (0.84–1.66)	1.06 (0.74–1.52)	0.98 (0.67–1.45)
Baseline upper tertile TyG	1.17 (0.84–1.63)	0.92 (0.64–1.32)	0.84 (0.57–1.24)
Baseline upper tertile TyG‐BMI	1.19 (0.85–1.66)	0.91 (0.63–1.31)	0.89 (0.62–1.28)
Mean 1st year continuous AIP	1.13 (0.96–1.32)	1.00 (0.84–1.19)	0.88 (0.73–1.07)
Mean 1st year continuous TyG	1.34 (1.14–1.58)***	1.09 (0.91–1.30)	0.97 (0.80–1.17)
Mean 1st year continuous TyG‐BMI	1.16 (0.99–1.35)	0.96 (0.81–1.13)	0.92 (0.78–1.08)
Mean 1st year upper tertile AIP	1.09 (0.77–1.54)	0.90 (0.64–1.27)	0.86 (0.61–1.21)
Mean 1st year upper tertile TyG	1.61 (1.14–2.27)**	1.06 (0.72–1.54)	0.79 (0.52–1.21)
Mean 1st year upper tertile TyG‐BMI	1.32 (0.93–1.88)	0.91 (0.63–1.33)	0.82 (0.56–1.19)
Major adverse cardiovascular events (*n* = 172)			
Baseline continuous AIP	1.06 (0.89–1.26)	1.04 (0.86–1.26)	1.05 (0.85–1.28)
Baseline continuous TyG	1.00 (0.84–1.19)	0.91 (0.76–1.10)	0.89 (0.73–1.09)
Baseline continuous TyG‐BMI	0.98 (0.82–1.16)	0.87 (0.72–1.05)	0.87 (0.72–1.05)
Baseline upper tertile AIP	1.12 (0.77–1.64)	1.09 (0.73–1.63)	1.08 (0.71–1.66)
Baseline upper tertile TyG	1.08 (0.75–1.56)	0.91 (0.62–1.36)	0.89 (0.58–1.37)
Baseline upper tertile TyG‐BMI	1.08 (0.75–1.57)	0.86 (0.58–1.29)	0.86 (0.58–1.29)
Mean 1st year continuous AIP	1.15 (0.96–1.37)	1.06 (0.87–1.28)	0.99 (0.80–1.22)
Mean 1st year continuous TyG	1.30 (1.09–1.55)**	1.07 (0.88–1.31)	1.00 (0.80–1.23)
Mean 1st year continuous TyG‐BMI	1.10 (0.92–1.30)	0.90 (0.75–1.08)	0.87 (0.72–1.05)
Mean 1st year upper tertile AIP	1.14 (0.78–1.67)	0.92 (0.61–1.37)	0.79 (0.51–1.22)
Mean 1st year upper tertile TyG	1.55 (1.06–2.26)*	1.06 (0.70–1.62)	0.88 (0.55–1.40)
Mean 1st year upper tertile TyG‐BMI	1.20 (0.82–1.77)	0.82 (0.55–1.24)	0.76 (0.50–1.15)
Cardiovascular mortality (*n* = 127)			
Baseline continuous AIP	1.08 (0.88–1.32)	1.08 (0.87–1.35)	1.11 (0.88–1.41)
Baseline continuous TyG	1.02 (0.83–1.25)	1.01 (0.82–1.24)	1.03 (0.82–1.30)
Baseline continuous TyG‐BMI	1.09 (0.89–1.33)	1.00 (0.80–1.23)	1.00 (0.80–1.24)
Baseline upper tertile AIP	1.17 (0.76–1.81)	1.17 (0.74–1.84)	1.24 (0.80–1.92)
Baseline upper tertile TyG	1.20 (0.78–1.84)	1.15 (0.73–1.80)	1.23 (0.75–2.01)
Baseline upper tertile TyG‐BMI	1.37 (0.89–2.13)	1.19 (0.74–1.89)	1.20 (0.75–1.93)
Mean 1st year continuous AIP	1.14 (0.92–1.40)	1.04 (0.83–1.30)	1.01 (0.80–1.29)
Mean 1st year continuous TyG	1.32 (1.08–1.62)**	1.05 (0.84–1.32)	1.03 (0.80–1.33)
Mean 1st year continuous TyG‐BMI	1.27 (1.05–1.55)*	1.06 (0.86–1.32)	1.06 (0.85–1.31)
Mean 1st year upper tertile AIP	1.15 (0.74–1.77)	0.93 (0.59–1.47)	0.89 (0.55–1.45)
Mean 1st year upper tertile TyG	1.54 (1.01–2.36)*	0.95 (0.59–1.53)	0.87 (0.51–1.48)
Mean 1st year upper tertile TyG‐BMI	1.54 (0.98–2.40)	1.08 (0.67–1.74)	1.06 (0.65–1.71)
All‐cause mortality (*n* = 263)			
Baseline continuous AIP	1.06 (0.92–1.22)	1.09 (0.94–1.27)	1.14 (0.97–1.34)
Baseline continuous TyG	1.09 (0.94–1.26)	1.05 (0.90–1.23)	1.10 (0.93–1.31)
Baseline continuous TyG‐BMI	1.05 (0.91–1.21)	0.96 (0.83–1.12)	0.97 (0.84–1.13)
Baseline upper tertile AIP	1.09 (0.81–1.48)	1.14 (0.83–1.57)	1.23 (0.88–1.72)
Baseline upper tertile TyG	1.29 (0.95–1.74)	1.21 (0.88–1.68)	1.34 (0.94–1.89)
Baseline upper tertile TyG‐BMI	1.28 (0.94–1.73)	1.09 (0.79–1.49)	1.11 (0.81–1.53)
Mean 1st year continuous AIP	1.13 (0.97–1.30)	1.09 (0.93–1.27)	1.09 (0.92–1.28)
Mean 1st year continuous TyG	1.27 (1.10–1.47)***	1.12 (0.95–1.31)	1.13 (0.95–1.35)
Mean 1st year continuous TyG‐BMI	1.11 (0.97–1.28)	0.94 (0.81–1.09)	0.93 (0.80–1.09)
Mean 1st year upper tertile AIP	1.11 (0.82–1.51)	1.00 (0.72–1.38)	0.99 (0.71–1.39)
Mean 1st year upper tertile TyG	1.43 (1.05–1.94)*	1.07 (0.77–1.50)	1.06 (0.73–1.55)
Mean 1st year upper tertile TyG‐BMI	1.12 (0.82–1.52)	0.81 (0.58–1.12)	0.80 (0.57–1.11)

*Note*: Values are adjusted hazard ratios (95% confidence intervals); ***p < 0.001, **p < 0.01, *p < 0.05.

Abbreviations: AIP, atherogenic index of plasma; CI, confidence interval; HR, hazard ratio; TyG, triglyceride‐glucose index; TyG‐BMI, triglyceride‐glucose‐body mass index.

^a^ Model 1 is adjusted for age and sex; Model 2 is further adjusted for BMI (except in analyses of TyG‐BMI index), smoking, physical activity, diabetes duration, pre‐existent macro‐ and microvascular complications, baseline SBP and HbA_1c_, number of anti‐hypertensive drugs in use, and use of insulin, statins, fibrates and aspirin; and Model 3 is further adjusted for serum LDL‐cholesterol. In analyses with mean 1st year triglyceride parameters, BMI, SBP, HbA_1c_ and LDL‐cholesterol were also updated to mean 1st year values.

Table 5 depicts the same multivariable analyses for microvascular outcomes. Similar to macrovascular/mortality outcomes, no triglyceride‐derived metabolic parameter was significantly associated with higher risks of microvascular complication development, most yet after simple adjustment for age/sex (Model 1). Only for retinopathy development/worsening outcome, TyG measured as mean values during the 1st year of follow‐up was associated with a significant 24% (HR: 1.24; 95% CI: 1.01–1.53) higher risk (for increment of 1‐SD in analysis as a continuous variable) and with a 75% (HR: 1.75; 95% CI: 1.11–2.78) higher risk (when compared the upper with the lower tertile subgroup), after multivariable adjustments for traditional risk factors (Model 2). However, these associations attenuated and became non‐significant after further adjustment for mean 1st year serum LDL‐ or total cholesterol levels (HR: 1.16; 95% CI: 0.92–1.45, and HR: 1.55; 95% CI: 0.95–2.53, respectively, in continuous and tertile analyses, Model 3).

**TABLE 5:** Adjusted risks associated with triglyceride‐derived metabolic parameters (continuous and categorical), measured at baseline and during the first year of follow‐up, for microvascular outcomes in 667 individuals with type 2 diabetes.

Outcomes	Model 1^a^	Model 2^a^	Model 3^a^
Triglyceride‐derived metabolic parameters	HR (95% CI)	HR (95% CI)	HR (95% CI)
Advanced renal failure (*n* = 98)			
Baseline continuous AIP	1.20 (0.95–1.51)	1.11 (0.85–1.44)	1.10 (0.84–1.44)
Baseline continuous TyG	1.31 (1.04–1.65)*	1.07 (0.83–1.38)	1.05 (0.80–1.38)
Baseline continuous TyG‐BMI	1.14 (0.91–1.42)	1.01 (0.80–1.27)	1.00 (0.79–1.27)
Baseline upper tertile AIP	1.24 (0.78–1.98)	1.07 (0.63–1.79)	1.05 (0.61–1.80)
Baseline upper tertile TyG	1.60 (0.99–2.58)	1.03 (0.60–1.78)	0.99 (0.56–1.76)
Baseline upper tertile TyG‐BMI	1.23 (0.75–2.02)	0.96 (0.57–1.60)	0.94 (0.56–1.58)
Mean 1st year continuous AIP	1.28 (1.02–1.62)*	1.13 (0.88–1.45)	1.04 (0.80–1.36)
Mean 1st year continuous TyG	1.41 (1.13–1.76)**	1.08 (0.84–1.38)	0.97 (0.74–1.26)
Mean 1st year continuous TyG‐BMI	1.21 (0.97–1.51)	0.99 (0.78–1.25)	0.94 (0.74–1.19)
Mean 1st year upper tertile AIP	1.52 (0.95–2.43)	1.15 (0.69–1.94)	1.03 (0.60–1.76)
Mean 1st year upper tertile TyG	1.89 (1.14–3.12)*	1.11 (0.63–1.96)	0.88 (0.48–1.61)
Mean 1st year upper tertile TyG‐BMI	1.37 (0.84–2.23)	0.93 (0.56–1.55)	0.83 (0.49–1.39)
Microalbuminuria development (*n* = 124)			
Baseline continuous AIP	0.92 (0.75–1.13)	0.91 (0.73–1.15)	0.96 (0.75–1.22)
Baseline continuous TyG	0.91 (0.75–1.11)	0.84 (0.67–1.05)	0.87 (0.68–1.11)
Baseline continuous TyG‐BMI	0.93 (0.76–1.14)	0.87 (0.70–1.07)	0.88 (0.71–1.10)
Baseline upper tertile AIP	0.78 (0.50–1.20)	0.76 (0.47–1.22)	0.82 (0.49–1.36)
Baseline upper tertile TyG	0.95 (0.62–1.45)	0.85 (0.53–1.35)	0.92 (0.56–1.51)
Baseline upper tertile TyG‐BMI	0.91 (0.57–1.45)	0.76 (0.47–1.25)	0.78 (0.48–1.28)
Mean 1st year continuous AIP	0.97 (0.79–1.19)	0.93 (0.75–1.17)	0.99 (0.78–1.26)
Mean 1st year continuous TyG	1.02 (0.84–1.24)	0.90 (0.72–1.12)	0.95 (0.74–1.22)
Mean 1st year continuous TyG‐BMI	0.98 (0.80–1.19)	0.92 (0.75–1.14)	0.94 (0.76–1.17)
Mean 1st year upper tertile AIP	0.82 (0.53–1.27)	0.76 (0.48–1.22)	0.84 (0.51–1.38)
Mean 1st year upper tertile TyG	1.07 (0.70–1.63)	0.88 (0.55–1.41)	1.00 (0.59–1.69)
Mean 1st year upper tertile TyG‐BMI	0.85 (0.53–1.37)	0.74 (0.45–1.21)	0.77 (0.47–1.27)
Retinopathy development/worsening (*n* = 154)			
Baseline continuous AIP	1.02 (0.85–1.23)	1.03 (0.85–1.26)	0.99 (0.81–1.22)
Baseline continuous TyG	1.11 (0.92–1.33)	0.96 (0.78–1.18)	0.90 (0.72–1.12)
Baseline continuous TyG‐BMI	0.97 (0.81–1.15)	0.98 (0.82–1.18)	0.97 (0.80–1.16)
Baseline upper tertile AIP	0.98 (0.66–1.46)	1.01 (0.66–1.56)	0.92 (0.59–1.45)
Baseline upper tertile TyG	1.44 (0.97–2.14)	1.07 (0.69–1.65)	0.98 (0.62–1.53)
Baseline upper tertile TyG‐BMI	0.96 (0.64–1.45)	0.99 (0.65–1.51)	0.98 (0.64–1.49)
Mean 1st year continuous AIP	1.10 (0.92–1.33)	1.09 (0.89–1.32)	1.02 (0.83–1.26)
Mean 1st year continuous TyG	1.36 (1.14–1.63)***	1.24 (1.01–1.53)*	1.16 (0.92–1.45)
Mean 1st year continuous TyG‐BMI	1.14 (0.95–1.36)	1.10 (0.91–1.32)	1.07 (0.89–1.29)
Mean 1st year upper tertile AIP	1.25 (0.85–1.84)	1.14 (0.75–1.73)	1.00 (0.64–1.55)
Mean 1st year upper tertile TyG	2.13 (1.40–3.24)***	1.75 (1.11–2.78)*	1.55 (0.95–2.53)
Mean 1st year upper tertile TyG‐BMI	1.35 (0.88–2.07)	1.31 (0.84–2.05)	1.25 (0.80–1.95)
Peripheral neuropathy development/worsening (*n* = 173)			
Baseline continuous AIP	1.00 (0.85–1.19)	0.97 (0.81–1.16)	1.09 (0.90–1.33)
Baseline continuous TyG	1.15 (0.97–1.36)	0.97 (0.80–1.16)	1.13 (0.92–1.39)
Baseline continuous TyG‐BMI	1.11 (0.95–1.30)	1.03 (0.87–1.23)	1.09 (0.91–1.30)
Baseline upper tertile AIP	1.04 (0.72–1.50)	0.98 (0.66–1.46)	1.28 (0.83–1.99)
Baseline upper tertile TyG	1.41 (0.98–2.03)	1.05 (0.71–1.55)	1.40 (0.92–2.13)
Baseline upper tertile TyG‐BMI	1.34 (0.91–1.96)	1.11 (0.74–1.67)	1.18 (0.78–1.77)
Mean 1st year continuous AIP	1.05 (0.88–1.24)	0.99 (0.83–1.19)	1.10 (0.90–1.34)
Mean 1st year continuous TyG	1.26 (1.06–1.49)**	1.08 (0.89–1.30)	1.10 (0.91–1.33)
Mean 1st year continuous TyG‐BMI	1.17 (1.00–1.37)*	1.04 (0.87–1.25)	1.09 (0.91–1.30)
Mean 1st year upper tertile AIP	1.09 (0.70–1.46)	0.91 (0.61–1.34)	1.01 (0.72–1.67)
Mean 1st year upper tertile TyG	1.56 (1.09–2.23)*	1.07 (0.72–1.60)	1.13 (0.75–1.69)
Mean 1st year upper tertile TyG‐BMI	1.64 (1.11–2.42)*	1.40 (0.92–2.14)	1.40 (0.91–2.15)

*Note*: Values are adjusted hazard ratios (95% confidence intervals); ***p < 0.001, **p < 0.01, *p < 0.05.

Abbreviations: AIP, atherogenic index of plasma; CI, confidence interval; HR, hazard ratio; TyG, triglyceride‐glucose index; TyG‐BMI, triglyceride‐glucose‐body mass index.

^a^ Model 1 is adjusted for age and sex; Model 2 is further adjusted for BMI (except in analyses of TyG‐BMI index, and in neuropathy analyses, body height was used instead of BMI), smoking, physical activity, diabetes duration, pre‐existing macro‐ and microvascular complications, baseline SBP and HbA_1c_, number of anti‐hypertensive drugs in use, and use of insulin, statins, fibrates and aspirin (analyses of renal outcomes were further adjusted for baseline eGFR and albuminuria); and Model 3 is further adjusted for serum LDL‐cholesterol. In analyses with mean 1st year triglyceride parameters, BMI, SBP, HbA_1c_ and LDL‐cholesterol were also updated to mean 1st year values.

## 4 DISCUSSION

This prospective long‐term cohort study demonstrated that no triglyceride‐derived metabolic parameter, neither AIP nor TyG and its associated body adiposity indices, was a predictor of adverse outcomes, either micro‐ or macrovascular endpoints or of mortality, in individuals with long‐standing type 2 diabetes. Indeed, most of the non‐significant associations were already demonstrated after simple sex and age adjustments, which became even more evident after increasing statistical adjustments for traditional risk factors and serum cholesterol levels. Therefore, from a clinical standpoint, our findings suggest that these triglyceride‐derived metabolic indices should not be used to improve risk stratification in people with type 2 diabetes.

Although many previous cohort studies in general population samples and meta‐analyses of these studies^9^, ^10^, ^11^, ^12^ indicated that the triglyceride‐derived metabolic parameters might be predictors of type 2 diabetes incidence and of coronary disease events development in general populations, mostly from the Asian region, the evidence in individuals with type 2 diabetes is more scarce and controversial. Some previous studies in patients with type 2 diabetes enrolled individuals with already established cardiovascular diseases, such as heart failure,^24^ acute myocardial infarction^25^ and coronary heart disease.^26^ Moreover, most previous studies were also from the Asian region,^27^ turning uncertain their generalizability to non‐Asian populations with type 2 diabetes. Indeed, as far as we know, there were only five longitudinal studies in non‐Asian individuals with type 2 diabetes.^6^, ^13^, ^14^, ^15^, ^16^ Two of them were post‐hoc analyses of randomized trials, the ACCORD^13^ and the Look AHEAD^14^ trials. In the ACCORD data analysis, with 10,251 high‐risk individuals with type 2 diabetes, a high AIP was a predictor of MACEs and myocardial infarctions, but not of strokes and all‐cause mortality.^13^ In the Look AHEAD data analysis, with 4199 individuals with type 2 diabetes and obesity/overweight, only low serum HDL‐cholesterol levels, but not high serum triglyceride levels, were associated with higher risks of total CVEs and coronary events, while the risks associated with the presence of metabolic dyslipidaemia (the combination of low HDL‐cholesterol and high triglycerides, hence equivalent to AIP) were identical to those of low HDL‐cholesterol isolated, indicating that all prognostic information came from HDL‐cholesterol, not from serum triglyceride levels.^14^ Otherwise, in an analysis of the NHANES data from 2001 to 2018, with 1072 individuals with prediabetes or type 2 diabetes with pre‐existent cardiovascular diseases,^15^ the continuous TyG had a significant non‐linear association with higher risks of all‐cause and cardiovascular mortality; whereas in quartile analyses, only the 2nd quartile subgroup had a lower risk of mortality, while the 3rd and 4th quartile subgroups had no excess risks in relation to the 1st quartile subgroup. Beyond including individuals with prediabetes and all participants with cardiovascular diseases, the researchers did not adjust their analysis for several important confounders, such as diabetes duration, physical activity, serum cholesterol and HbA_1c_ levels, turning difficult the comparison with other investigations in type 2 diabetes. A smaller cohort of 568 individuals with type 2 diabetes from Italy^16^ demonstrated that the TyG was associated with a higher risk of all‐cause mortality, but without adjustment for systolic BP, diabetes duration and physical activity. Finally, an analysis of the UK Biobank database, with 374,274 participants without diabetes or cardiovascular diseases at baseline,^6^ demonstrated that the AIP and the TyG were both significant strong predictors of diabetes development; however, once type 2 diabetes was diagnosed (in 13,030 individuals), these indices did not predict further progression to cardiovascular diseases, either coronary or cerebrovascular diseases development. Our findings regarding the lack of any prognostic importance of triglyceride‐derived metabolic parameters for cardiovascular or mortality are concordant to the results reported by the Look AHEAD trial^14^ and from the UK Biobank database,^6^ but discordant to the analysis of the ACCORD trial.^13^ Possibly, the 2 × 2 design of the ACCORD trial, with randomized arms of intensive and conventional BP and HbA_1c_ levels, might explain at least partially these disparities, since the investigators did not adjust their analyses to randomization arms. Moreover, it was a post‐hoc analysis, not a prespecified one, which also limits its usefulness. Overall, our findings support that these triglyceride‐derived metabolic parameters are not useful for cardiovascular/mortality risk stratification in non‐Asian individuals with type 2 diabetes.

Regarding the associations between triglyceride‐derived parameters with risks of microvascular complications development in type 2 diabetes, there were only 2 previous longitudinal studies that addressed this issue. The first one^17^ was a cohort from China that evaluated 2943 (for renal outcome) to 3360 (for retinopathy) individuals with type 2 diabetes followed up for 2 years. It demonstrated that a high baseline AIP was associated with higher risk of developing chronic kidney disease (CKD), defined as an eGFR <60 mL/min/1.73m^2^ or a urinary albumin/creatinine ratio ≥3.39 mg/mmol, but not of developing diabetic retinopathy (DR), after adjustments for classic risk factors including systolic BP, HbA_1c_ and serum LDL‐cholesterol.^17^ The second one^18^ was a post‐hoc analysis of the ACCORD trial data, with 5000 (for neuropathy) to 6503 (for retinopathy) participants with type 2 diabetes followed up for 3.7 years. It demonstrated that baseline AIP and TyG were both associated with higher risks of developing CKD (defined as doubling of serum creatinine, or a >20 mL/min/1.73 m^2^ decrease in eGFR, or a serum creatinine >3.3 mg/dL, or end‐stage renal disease development, or new microalbuminuria development) and of peripheral neuropathy (detected by the Michigan Neuropathy Screening Instrument or by physical examination), but not of developing DR. However, beyond not being a prespecified analysis of the ACCORD trial, the authors did not adjust their analyses to randomization arms, diabetes duration, and serum HbA_1c_ and cholesterol levels.^18^ Our study findings are discordant with these previous two studies,^17^, ^18^ possibly because of different populations (in the case of the Chinese cohort), definition of microvascular endpoints, duration of follow‐up and insufficient statistical adjustments (in the case of the ACCORD analysis). Our study had the longest follow‐up, predefined standardized microvascular endpoints assessment and adjudication, and the most complete statistical adjustment. We observed that mean 1st year TyG was a significant predictor of DR development/worsening in Model 2 (full adjustment without other lipid parameters), but this association became non‐significant after further adjustments for LDL‐ or total cholesterol (Model 3). This suggests that the strongest lipid parameter associated with DR is actually serum cholesterol (total or LDL‐), as we previously reported,^20^ but not any triglyceride‐derived parameter.

This study has some limitations that are warrant to discuss. First, as in all observational cohort studies, unmeasured or unknown factors might still have influenced the results and some residual confounding could not be ruled out. Moreover, no causal relationships, nor physiopathological deductions, can be made, but only hypothesized. Second, it is a single‐centre cohort, and validation from other cohorts, ideally multi‐centre and from non‐Asian populations, is clearly needed. Furthermore, this cohort mainly enrolled middle‐aged to elderly individuals with long‐standing type 2 diabetes followed up in a tertiary‐care University hospital. Hence, our results might not be generalized to younger individuals with recent‐onset type 2 diabetes or at primary care management. On the other hand, the main strength of this study is its well‐documented cohort of individuals with type 2 diabetes under standardized care and regular outcomes evaluation over a long 11‐year follow‐up period.

In conclusion, this prospective long‐term cohort study did not find evidence that any triglyceride‐derived metabolic parameters, AIP or TyG, can predict adverse outcomes, micro‐ or macrovascular complications or mortality, in individuals with type 2 diabetes. In this way, our findings suggest that these metabolic parameters should not be recommended to refine risk stratification for cardiovascular or microvascular complications development in clinical type 2 diabetes management, particularly in non‐Asian populations.

## FUNDING INFORMATION

This study was supported by grants from the Conselho Nacional de Desenvolvimento Científico e Tecnológico (CNPq, Brazil) and from the Fundação Carlos Chagas Filho de Amparo a Pesquisa do Estado do Rio de Janeiro (FAPERJ, Brazil).

## CONFLICT OF INTEREST STATEMENT

None to declare.

## Supporting information


Data S1.

## Data Availability

The data that support the findings of this study are available upon request from the corresponding author.

## References

[1] American Diabetes Association Professional Practice Committee . 10. Cardiovascular disease and risk management: standards of Care in Diabetes‐2025. Diabetes Care. 2025;48(1 Suppl 1):S207‐S238. doi:10.2337/dc25-S010 39651970 PMC11635050

[2] American Diabetes Association Professional Practice Committee . 11. Chronic kidney disease and risk management: standards of Care in Diabetes‐2025. Diabetes Care. 2025;48(1 Suppl 1):S239‐S251. doi:10.2337/dc25-S011 39651975 PMC11635029

[3] American Diabetes Association Professional Practice Committee . 12. Retinopathy, neuropathy, and foot care: standards of Care in Diabetes‐2025. Diabetes Care. 2025;48(1 Suppl 1):S252‐S265. doi:10.2337/dc25-S012 39651973 PMC11635040

[4] NCD Risk Factor Collaboration (NCD‐RisC) . Worldwide trends in diabetes prevalence and treatment from 1990 to 2022: a pooled analysis of 1108 population‐representative studies with 141 million participants. Lancet. 2024;404(10467):2077‐2093. doi:10.1016/S0140-6736(24)02317-1 39549716 PMC7616842

[5] Lee SH , Park SY , Choi CS . Insulin resistance: from mechanisms to therapeutic strategies. Diabetes Metab J. 2022;46(1):15‐37. doi:10.4093/dmj.2021.0280 34965646 PMC8831809

[6] Tian Z , Yang L , Li Y , Huang Y , Yang J , Xue F . Associations of different insulin resistance‐related indices with the incidence and progression trajectory of cardiometabolic multimorbidity: a prospective cohort study from UK biobank. Cardiovasc Diabetol. 2025;24(1):257. doi:10.1186/s12933-025-02819-0 40533754 PMC12175334

[7] Dobiásová M , Frohlich J . The plasma parameter log (TG/HDL‐C) as an atherogenic index: correlation with lipoprotein particle size and esterification rate in apoB‐lipoprotein‐depleted plasma (FER(HDL)). Clin Biochem. 2001;34(7):583‐588. doi:10.1016/s0009-9120(01)00263-6 11738396

[8] Guerrero‐Romero F , Simental‐Mendía LE , González‐Ortiz M , et al. The product of triglycerides and glucose, a simple measure of insulin sensitivity. Comparison with the euglycemic‐hyperinsulinemic clamp. J Clin Endocrinol Metab. 2010;95(7):3347‐3351. doi:10.1210/jc.2010-0288 20484475

[9] Liu X , Tan Z , Huang Y , et al. Relationship between the triglyceride‐glucose index and risk of cardiovascular diseases and mortality in the general population: a systematic review and meta‐analysis. Cardiovasc Diabetol. 2022;21(1):124. doi:10.1186/s12933-022-01546-0 35778731 PMC9250255

[10] Chen Y , Chang Z , Liu Y , et al. Triglyceride to high‐density lipoprotein cholesterol ratio and cardiovascular events in the general population: a systematic review and meta‐analysis of cohort studies. Nutr Metab Cardiovasc Dis. 2022;32(2):318‐329. doi:10.1016/j.numecd.2021.11.005 34953633

[11] Nayak SS , Kuriyakose D , Polisetty LD , et al. Diagnostic and prognostic value of triglyceride glucose index: a comprehensive evaluation of meta‐analysis. Cardiovasc Diabetol. 2024;23(1):310. doi:10.1186/s12933-024-02392-y 39180024 PMC11344391

[12] Rao X , Xin Z , Yu Q , et al. Triglyceride‐glucose‐body mass index and the incidence of cardiovascular diseases: a meta‐analysis of cohort studies. Cardiovasc Diabetol. 2025;24(1):34. doi:10.1186/s12933-025-02584-0 39844258 PMC11756031

[13] Fu L , Zhou Y , Sun J , et al. Atherogenic index of plasma is associated with major adverse cardiovascular events in patients with type 2 diabetes mellitus. Cardiovasc Diabetol. 2021;20(1):201. doi:10.1186/s12933-021-01393-5 34610830 PMC8493717

[14] Kaze AD , Santhanam P , Musani SK , Ahima R , Echouffo‐Tcheugui JB . Metabolic dyslipidemia and cardiovascular outcomes in type 2 diabetes mellitus: findings from the look AHEAD study. J Am Heart Assoc. 2021;10(7):e016947. doi:10.1161/JAHA.120.016947 33728932 PMC8174364

[15] Zhang Q , Xiao S , Jiao X , Shen Y . The triglyceride‐glucose index is a predictor for cardiovascular and all‐cause mortality in CVD patients with diabetes or pre‐diabetes: evidence from NHANES 2001‐2018. Cardiovasc Diabetol. 2023;22(1):279. doi:10.1186/s12933-023-02030-z 37848879 PMC10583314

[16] Sbriscia M , Colombaretti D , Giuliani A , et al. Triglyceride glucose index predicts long‐term mortality and major adverse cardiovascular events in patients with type 2 diabetes. Cardiovasc Diabetol. 2025;24(1):115. doi:10.1186/s12933-025-02671-2 40065340 PMC11895143

[17] Zhang J , Liu C , Peng Y , et al. Impact of baseline and trajectory of the atherogenic index of plasma on incident diabetic kidney disease and retinopathy in participants with type 2 diabetes: a longitudinal cohort study. Lipids Health Dis. 2024;23(1):11. doi:10.1186/s12944-024-02003-5 38212770 PMC10782533

[18] Cao BF , Liu K , Chen HW , et al. Impact of baseline and trajectory of the cardiometabolic indices on incident microvascular complications in patients with type 2 diabetes. Atherosclerosis. 2025;407:120407. doi:10.1016/j.atherosclerosis.2025.120407 40561674

[19] Cardoso CR , Moran CB , Marinho FS , Ferreira MT , Salles GF . Increased aortic stiffness predicts future development and progression of peripheral neuropathy in patients with type 2 diabetes: the Rio de Janeiro type 2 diabetes cohort study. Diabetologia. 2015;58(9):2161‐2168. doi:10.1007/s00125-015-3658-9 26044207

[20] Cardoso CRL , Leite NC , Dib E , Salles GF . Predictors of development and progression of retinopathy in patients with type 2 diabetes: importance of blood pressure parameters. Sci Rep. 2017;7(1):4867. doi:10.1038/s41598-017-05159-6 28687808 PMC5501788

[21] Cardoso CRL , Leite NC , Salles GC , Ferreira MT , Salles GF . Aortic stiffness and ambulatory blood pressure as predictors of diabetic kidney disease: a competing risks analysis from the Rio de Janeiro type 2 diabetes cohort study. Diabetologia. 2018;61(2):455‐465. doi:10.1007/s00125-017-4484-z 29063128

[22] Cardoso CRL , da Silva Pereira L , Leite NC , Salles GF . Prognostic importance of baseline and changes in serum uric acid for macro/microvascular and mortality outcomes in individuals with type 2 diabetes: the Rio de Janeiro type 2 diabetes cohort. J Diabetes Complicat. 2025;39(1):108921. doi:10.1016/j.jdiacomp.2024.108921 39616659

[23] Cardoso CRL , Leite NC , de Souza AC , Paiva TRA , Salles GC , Salles GF . Comparison of the prognostic value of different arterial sites atherosclerosis risk markers for development of macro‐ and microvascular complications in individuals with type 2 diabetes: the Rio de Janeiro type 2 diabetes cohort study. Diabetes Res Clin Pract. 2025;226:112322. doi:10.1016/j.diabres.2025.112322 40499685

[24] Guo W , Zhao L , Mo F , et al. The prognostic value of the triglyceride glucose index in patients with chronic heart failure and type 2 diabetes: a retrospective cohort study. Diabetes Res Clin Pract. 2021;177:108786. doi:10.1016/j.diabres.2021.108786 33812901

[25] Zhang Y , Ding X , Hua B , et al. Predictive effect of triglyceride‐glucose index on clinical events in patients with type 2 diabetes mellitus and acute myocardial infarction: results from an observational cohort study in China. Cardiovasc Diabetol. 2021;20(1):43. doi:10.1186/s12933-021-01236-3 33573649 PMC7879620

[26] Tao S , Yu L , Li J , et al. Multiple triglyceride‐derived metabolic indices and incident cardiovascular outcomes in patients with type 2 diabetes and coronary heart disease. Cardiovasc Diabetol. 2024;23(1):359. doi:10.1186/s12933-024-02446-1 39402572 PMC11472491

[27] Zhang J , Zhan Q , Deng Z , et al. Does diabetes modify the triglyceride‐glucose index associated with cardiovascular events and mortality? A meta‐analysis of 50 cohorts involving 7,239,790 participants. Cardiovasc Diabetol. 2025;24(1):42. doi:10.1186/s12933-025-02585-z 39871273 PMC11773825

