# Supplementary material for: Prognostic value of triglyceride‐derived metabolic parameters for micro‐ and macrovascular complications and mortality in individuals with type 2 diabetes: The Rio de Janeiro type 2 diabetes cohort study
Source: Diabet Med. 2026 Feb 19;43(4):e70263. doi: 10.1111/dme.70263 (PMC12982650; doi:10.1111/dme.70263)
Supplement: Supplementary file 1 — Data S1. [file DME-43-e70263-s001.docx]

**Supplementary material**

**Title:** Prognostic Value of Triglyceride-Derived Metabolic Parameters for Micro- and Macrovascular Complications and Mortality in Individuals with Type 2 Diabetes: The Rio de Janeiro Type 2 Diabetes Cohort Study.

**Authors:** Claudia R L Cardoso; Guilherme P Castro; Nathalie C Leite; Gil F Salles

**Methods**

**Cohort overview and baseline protocol**

This was a prospective longitudinal observational cohort study with 692 individuals with type 2 diabetes, enrolled between 2004 and 2008 and regularly followed-up until 2019 in the diabetes outclinic of our tertiary-care University Hospital. All participants gave written informed consent, and the local Ethics Committee had previously approved the study protocol. The characteristics of this cohort, the baseline procedures and the diagnostic definitions have been described previously.^1-5^ In summary, inclusion criteria were all adult individuals with type 2 diabetes up to 80 years old with either any microvascular (retinopathy, nephropathy or neuropathy) or macrovascular (coronary, cerebrovascular or peripheral artery disease) complication, or with at least two other modifiable cardiovascular risk factors (hypertension, dyslipidemia or smoking). Exclusion criteria were morbid obesity (body mass index [BMI] >40 kg/m^2^), advanced renal failure (serum creatinine >180 μmol/L or estimated glomerular filtration rate <30 ml/min/1.73m^2^) or the presence of any serious concomitant disease limiting life expectancy (such as severe class III or IV heart failure, Child-Pugh class B or C chronic liver diseases, symptomatic severe chronic lung diseases, advanced dementia or cancer). For this specific analysis of triglyceride-derived metabolic parameters, 25 individuals (4%) with baseline serum triglycerides ≥500 mg/dl, possibly indicating familial hypertriglyceridemia, were excluded, totaling 667 participants in this report. All were submitted to a standard baseline protocol that included a thorough clinical-laboratory evaluation. Diagnostic criteria for diabetic chronic complications were detailed previously.^1-5^ In brief, coronary heart disease was diagnosed by clinical, electrocardiographic criteria, or by positive ischemic stress tests. Cerebrovascular disease was diagnosed by history and physical examination, and peripheral arterial disease by an ankle-brachial index <0.9. The diagnosis of nephropathy needed at least two albuminurias ≥30mg/24h or confirmed reduction of glomerular filtration rate (eGFR ≤60 ml/min/1.73m^2^, estimated by the CKD-EPI equation, or serum creatinine >130 μmol/L). Peripheral neuropathy was determined by clinical examination (knee and ankle reflex activities, feet sensation with the Semmes-Weinstein monofilament, vibration with a 128-Hz tuning fork, pinprick and temperature sensations) and neuropathic symptoms were assessed by a standard validated questionnaire.^1^ Clinic blood pressure (BP) was measured three times using a digital oscillometric BP monitor (HEM-907XL, Omron Healthcare, Kyoto, Japan) with a suitable sized cuff on two occasions two weeks apart at study entry. The first measure of each visit was discarded and BP considered was the mean between the last two readings of each visit. Arterial hypertension was diagnosed if mean systolic (SBP) ≥140 mmHg or diastolic BP (DBP) ≥90 mmHg or if anti-hypertensive drugs had been prescribed. Laboratory evaluation included fasting glycemia, glycated hemoglobin (HbA_1c_), serum creatinine and lipids. Albuminuria was evaluated in two non-consecutive sterile 24-hour urine collections. All laboratory measurements at baseline and during follow-up were performed after a minimum 8-hour fasting by standardized automated methods (DAX 96 Bayer standards diagnosis) at the Central Laboratory of our University Hospital.

**Definition of retinopathy and peripheral neuropathy development/worsening during follow-up**

The presence and severity of diabetic retinopathy (DR) was ascertained at baseline and annually during follow-up by a complete ophthalmologic evaluation performed by a single experienced retinal specialist.^2^ It was graded according to the International Clinical Diabetic Retinopathy and Diabetic Macular Edema Disease scales^6^ into 5 stages: no retinopathy, mild non-proliferative retinopathy, moderate non-proliferative retinopathy, severe non-proliferative retinopathy and proliferative retinopathy. When there were inter-eye differences in DR severity, the eye with the severest DR was considered for DR classification. Incidence of DR was defined as having no DR signs in both eyes at baseline and having mild to severe non-proliferative DR or proliferative DR in either of the eyes at any annual examination. Progression of DR was defined as having mild non-proliferative DR at baseline and having severe non-proliferative DR, or proliferative DR or laser photocoagulation at any subsequent annual ophthalmologic examination; or as having moderate/severe non-proliferative DR at baseline and proliferative DR at any subsequent examination. All incident new DR and worsening DR cases were confirmed on a second ophthalmologic examination at least 3 months apart.^2^

For diabetic peripheral neuropathy (DPN) development/progression ascertainment, neuropathic symptoms were evaluated by a standard validated questionnaire,^7^ which allocates between 0 and 9 points to specific symptoms; and patients were classified as having no specific symptoms (0–2 points) or mild (3–4), moderate (5–6) or severe (7–9 points) symptoms. The clinical signs of peripheral neuropathy were assessed by the Neuropathy Disability Score (NDS)^7^ and by sensation in the feet tested with a Semmes-Weinstein 10 g/5.07 monofilament. The NDS combines examination of the ankle reflex with testing for vibration (with 128 Hz tuning fork), pinprick and temperature (with a cold tuning fork) sensations on both big toes, and gives a total score ranging from 0 (no signs) to 10 points. The sensory evaluations score either 0 (present) or 1 (reduced/absent), whereas the ankle reflex scores 0 (normal), 1 (present with reinforcement) or 2 (absent). Patients were classified as having mild neuropathic signs (3–5 points), moderate signs (6–8) or severe signs (9–10). In addition, sensation over the feet was tested with the Semmes-Weinstein monofilament on four plantar sites for each foot (the big toe and first, third and fifth metatarsal heads); if at least two insensitive regions were found, this was considered abnormal. All the examinations were repeated twice by the same independent observer, and in cases of disagreement a third examination was performed. The minimum criteria for the diagnosis of peripheral neuropathy were the presence of moderate symptoms (regardless of the presence of signs) or the presence of mild symptoms with moderate neuropathic signs. Hence, mild symptoms with or without mild signs were not considered adequate for establishing a diagnosis of peripheral neuropathy. Patients were examined by the same examiner at baseline and annually during follow-up. In those with DPN at baseline, the criteria for defining the progression of the neuropathy were as follows: an increase in the neuropathic symptom score from mild to moderate or from moderate to severe; an increase in the NDS from mild or moderate to severe; a doubling in the number of insensitive areas on monofilament examination (for those with at least two insensitive areas at the first assessment); and the occurrence of classic non-traumatic diabetic foot ulceration or toe amputation.^1^

**References**

1. Cardoso CR, Moran CB, Marinho FS, Ferreira MT, Salles GF. Increased aortic stiffness predicts future development and progression of peripheral neuropathy in patients with type 2 diabetes: the Rio de Janeiro Type 2 Diabetes Cohort Study. Diabetologia. 2015 Sep;58(9):2161-8. doi: 10.1007/s00125-015-3658-9.
2. Cardoso CRL, Leite NC, Dib E, Salles GF. Predictors of Development and Progression of Retinopathy in Patients with Type 2 Diabetes: Importance of Blood Pressure Parameters. Sci Rep. 2017 Jul 7;7(1):4867. doi: 10.1038/s41598-017-05159-6.
3. Cardoso CRL, Leite NC, Salles GC, Ferreira MT, Salles GF. Aortic stiffness and ambulatory blood pressure as predictors of diabetic kidney disease: a competing risks analysis from the Rio de Janeiro Type 2 Diabetes Cohort Study. Diabetologia. 2018 Feb;61(2):455-465. doi: 10.1007/s00125-017-4484-z.
4. Cardoso CRL, da Silva Pereira L, Leite NC, Salles GF. Prognostic importance of baseline and changes in serum uric acid for macro/microvascular and mortality outcomes in individuals with type 2 diabetes: The Rio de Janeiro type 2 diabetes cohort. J Diabetes Complications. 2025 Jan;39(1):108921. doi: 10.1016/j.jdiacomp.2024.108921.
5. Cardoso CRL, Leite NC, de Souza AC, Paiva TRA, Salles GC, Salles GF. Comparison of the prognostic value of different arterial sites atherosclerosis risk markers for development of Macro- and microvascular complications in individuals with type 2 diabetes: The Rio de Janeiro type 2 diabetes cohort study. Diabetes Res Clin Pract. 2025 Aug;226:112322. doi: 10.1016/j.diabres.2025.112322.
6. Wilkinson CP, Ferris FL 3rd, Klein RE, Lee PP, Agardh CD, Davis M, Dills D, Kampik A, Pararajasegaram R, Verdaguer JT; Global Diabetic Retinopathy Project Group. Proposed international clinical diabetic retinopathy and diabetic macular edema disease severity scales. Ophthalmology. 2003 Sep;110(9):1677-82. doi: 10.1016/S0161-6420(03)00475-5.
7. Young MJ, Boulton AJ, MacLeod AF, Williams DR, Sonksen PH. A multicentre study of the prevalence of diabetic peripheral neuropathy in the United Kingdom hospital clinic population. Diabetologia. 1993 Feb;36(2):150-4. doi: 10.1007/BF00400697.
